# International Journal of Pediatric Endocrinology reviewer acknowledgement 2015

**DOI:** 10.1186/s13633-016-0025-7

**Published:** 2016-02-24

**Authors:** Surendra Varma

**Affiliations:** Texas Tech University 3601 4th St, Lubbock, TX 79430 USA

## Abstract

The editor of *International Journal of Pediatric Endocrinology* would like to thank all of the reviewers who have contributed to the journal in 2015.

**Alan Rogol**

United States of America

**Alexander Jorge**

Brazil

**Alicia Romano**

United States of America

**Amanda Ackermann**

United States of America

**Ambika Ashraf**

United States of America

**Andrew Dauber**

United States of America

**Andrew Lane**

United States of America

**Archana Arya**

United States of America

**Arlan Rosenbloom**

United States of America

**Brenda Kohn**

United States of America

**Craig Alter**

United States of America

**Craig Jefferies**

New Zealand

**Daina Dreimane**

United States of America

**Daniel DeSalvo**

United States of America

**Dennis Styne**

United States of America

**Dorothy Shulman**

United States of America

**Edward Reiter**

United States of America

**Flavia Prodam**

Italy

**Grace Tannin**

United States of America

**Jan Idkowiak**

United Kingdom

**Jan-Maarten Wit**

Netherlands

**Jennifer Osipoff**

United States of America

**John Fuqua**

United States of America

**Jonathan Wasserman**

Canada

**Joshua Miller**

United States of America

**Kathleen Bethin**

United States of America

**Kimberly Tafuri**

United States of America

**Lefkothea Karaviti**

United States of America

**Liuska Pesce**

United States of America

**Maria Vogiatzi**

United States of America

**Mario Rotondi**

Italy

**Maya Lodish**

United States of America

**Mehul Dattani**

United Kingdom

**Meilan Rutter**

United States of America

**Munier Nour**

Canada

**Nicholas Jospe**

United States of America

**Olga Gupta**

United States of America

**Patricia Vuguin**

United States of America

**Paul Kaplowitz**

United States of America

**Paulo Collett-Solberg**

Brazil

**Philip Zeitler**

United States of America

**Robert Rosenfield**

United States of America

**Rubina Heptulla**

United States of America

**Sara DiVall**

United States of America

**Sara Watson**

United States of America

**Scott Rivkees**

United States of America

**Sungdae Moon**

South Korea

**Surendra Varma**

United States of America

**Susan Schuval**

United States of America

**Susanne Cabrera**

United States of America

**Theo Sas**

Netherlands

**Tracy Bekx**

United States of America

**Walter Bonfig**

Germany

**Zeynep Siklar**

Turkey

